# Measurement of the double-differential inclusive jet cross section in proton–proton collisions at $\sqrt{s} = 13\,\text {TeV}$

**DOI:** 10.1140/epjc/s10052-016-4286-3

**Published:** 2016-08-11

**Authors:** V. Khachatryan, A. M. Sirunyan, A. Tumasyan, W. Adam, E. Asilar, T. Bergauer, J. Brandstetter, E. Brondolin, M. Dragicevic, J. Erö, M. Flechl, M. Friedl, R. Frühwirth, V. M. Ghete, C. Hartl, N. Hörmann, J. Hrubec, M. Jeitler, A. König, I. Krätschmer, D. Liko, T. Matsushita, I. Mikulec, D. Rabady, N. Rad, B. Rahbaran, H. Rohringer, J. Schieck, J. Strauss, W. Treberer-Treberspurg, W. Waltenberger, C.-E. Wulz, V. Mossolov, N. Shumeiko, J. Suarez Gonzalez, S. Alderweireldt, E. A. De Wolf, X. Janssen, A. Knutsson, J. Lauwers, M. Van De Klundert, H. Van Haevermaet, P. Van Mechelen, N. Van Remortel, A. Van Spilbeeck, S. Abu Zeid, F. Blekman, J. D’Hondt, N. Daci, I. De Bruyn, K. Deroover, N. Heracleous, S. Lowette, S. Moortgat, L. Moreels, A. Olbrechts, Q. Python, S. Tavernier, W. Van Doninck, P. Van Mulders, I. Van Parijs, H. Brun, C. Caillol, B. Clerbaux, G. De Lentdecker, H. Delannoy, G. Fasanella, L. Favart, R. Goldouzian, A. Grebenyuk, G. Karapostoli, T. Lenzi, A. Léonard, J. Luetic, T. Maerschalk, A. Marinov, A. Randle-Conde, T. Seva, C. Vander Velde, P. Vanlaer, R. Yonamine, F. Zenoni, F. Zhang, A. Cimmino, T. Cornelis, D. Dobur, A. Fagot, G. Garcia, M. Gul, J. Mccartin, D. Poyraz, S. Salva, R. Schöfbeck, M. Tytgat, W. Van Driessche, E. Yazgan, N. Zaganidis, C. Beluffi, O. Bondu, S. Brochet, G. Bruno, A. Caudron, L. Ceard, S. De Visscher, C. Delaere, M. Delcourt, L. Forthomme, B. Francois, A. Giammanco, A. Jafari, P. Jez, M. Komm, V. Lemaitre, A. Magitteri, A. Mertens, M. Musich, C. Nuttens, K. Piotrzkowski, L. Quertenmont, M. Selvaggi, M. Vidal Marono, S. Wertz, N. Beliy, W. L. Aldá Júnior, F. L. Alves, G. A. Alves, L. Brito, M. Hamer, C. Hensel, A. Moraes, M. E. Pol, P. Rebello Teles, E. Belchior Batista Das Chagas, W. Carvalho, J. Chinellato, A. Custódio, E. M. Da Costa, G. G. Da Silveira, D. De Jesus Damiao, C. De Oliveira Martins, S. Fonseca De Souza, L. M. Huertas Guativa, H. Malbouisson, D. Matos Figueiredo, C. Mora Herrera, L. Mundim, H. Nogima, W. L. Prado Da Silva, A. Santoro, A. Sznajder, E. J. Tonelli Manganote, A. Vilela Pereira, S. Ahuja, C. A. Bernardes, S. Dogra, T. R. Fernandez Perez Tomei, E. M. Gregores, P. G. Mercadante, C. S. Moon, S. F. Novaes, Sandra S. Padula, D. Romero Abad, J. C. Ruiz Vargas, A. Aleksandrov, R. Hadjiiska, P. Iaydjiev, M. Rodozov, S. Stoykova, G. Sultanov, M. Vutova, A. Dimitrov, I. Glushkov, L. Litov, B. Pavlov, P. Petkov, W. Fang, M. Ahmad, J. G. Bian, G. M. Chen, H. S. Chen, M. Chen, Y. Chen, T. Cheng, R. Du, C. H. Jiang, D. Leggat, Z. Liu, F. Romeo, S. M. Shaheen, A. Spiezia, J. Tao, C. Wang, Z. Wang, H. Zhang, J. Zhao, C. Asawatangtrakuldee, Y. Ban, Q. Li, S. Liu, Y. Mao, S. J. Qian, D. Wang, Z. Xu, C. Avila, A. Cabrera, L. F. Chaparro Sierra, C. Florez, J. P. Gomez, C. F. González Hernández, J. D. Ruiz Alvarez, J. C. Sanabria, N. Godinovic, D. Lelas, I. Puljak, P. M. Ribeiro Cipriano, Z. Antunovic, M. Kovac, V. Brigljevic, D. Ferencek, K. Kadija, S. Micanovic, L. Sudic, A. Attikis, G. Mavromanolakis, J. Mousa, C. Nicolaou, F. Ptochos, P. A. Razis, H. Rykaczewski, M. Finger, M. Finger, E. Carrera Jarrin, S. Elgammal, A. Mohamed, Y. Mohammed, E. Salama, B. Calpas, M. Kadastik, M. Murumaa, L. Perrini, M. Raidal, A. Tiko, C. Veelken, P. Eerola, J. Pekkanen, M. Voutilainen, J. Härkönen, V. Karimäki, R. Kinnunen, T. Lampén, K. Lassila-Perini, S. Lehti, T. Lindén, P. Luukka, T. Peltola, J. Tuominiemi, E. Tuovinen, L. Wendland, J. Talvitie, T. Tuuva, M. Besancon, F. Couderc, M. Dejardin, D. Denegri, B. Fabbro, J. L. Faure, C. Favaro, F. Ferri, S. Ganjour, S. Ghosh, A. Givernaud, P. Gras, G. Hamel de Monchenault, P. Jarry, I. Kucher, E. Locci, M. Machet, J. Malcles, J. Rander, A. Rosowsky, M. Titov, A. Zghiche, A. Abdulsalam, I. Antropov, S. Baffioni, F. Beaudette, P. Busson, L. Cadamuro, E. Chapon, C. Charlot, O. Davignon, R. Granier de Cassagnac, M. Jo, S. Lisniak, P. Miné, I. N. Naranjo, M. Nguyen, C. Ochando, G. Ortona, P. Paganini, P. Pigard, S. Regnard, R. Salerno, Y. Sirois, T. Strebler, Y. Yilmaz, A. Zabi, J.-L. Agram, J. Andrea, A. Aubin, D. Bloch, J.-M. Brom, M. Buttignol, E. C. Chabert, N. Chanon, C. Collard, E. Conte, X. Coubez, J.-C. Fontaine, D. Gelé, U. Goerlach, A.-C. Le Bihan, J. A. Merlin, K. Skovpen, P. Van Hove, S. Gadrat, S. Beauceron, C. Bernet, G. Boudoul, E. Bouvier, C. A. Carrillo Montoya, R. Chierici, D. Contardo, B. Courbon, P. Depasse, H. El Mamouni, J. Fan, J. Fay, S. Gascon, M. Gouzevitch, G. Grenier, B. Ille, F. Lagarde, I. B. Laktineh, M. Lethuillier, L. Mirabito, A. L. Pequegnot, S. Perries, A. Popov, D. Sabes, V. Sordini, M. Vander Donckt, P. Verdier, S. Viret, A. Khvedelidze, D. Lomidze, C. Autermann, S. Beranek, L. Feld, A. Heister, M. K. Kiesel, K. Klein, M. Lipinski, A. Ostapchuk, M. Preuten, F. Raupach, S. Schael, C. Schomakers, J. F. Schulte, J. Schulz, T. Verlage, H. Weber, V. Zhukov, M. Brodski, E. Dietz-Laursonn, D. Duchardt, M. Endres, M. Erdmann, S. Erdweg, T. Esch, R. Fischer, A. Güth, T. Hebbeker, C. Heidemann, K. Hoepfner, S. Knutzen, M. Merschmeyer, A. Meyer, P. Millet, S. Mukherjee, M. Olschewski, K. Padeken, P. Papacz, T. Pook, M. Radziej, H. Reithler, M. Rieger, F. Scheuch, L. Sonnenschein, D. Teyssier, S. Thüer, V. Cherepanov, Y. Erdogan, G. Flügge, F. Hoehle, B. Kargoll, T. Kress, A. Künsken, J. Lingemann, A. Nehrkorn, A. Nowack, I. M. Nugent, C. Pistone, O. Pooth, A. Stahl, M. Aldaya Martin, I. Asin, K. Beernaert, O. Behnke, U. Behrens, A. A. Bin Anuar, K. Borras, A. Campbell, P. Connor, C. Contreras-Campana, F. Costanza, C. Diez Pardos, G. Dolinska, G. Eckerlin, D. Eckstein, E. Gallo, J. Garay Garcia, A. Geiser, A. Gizhko, J. M. Grados Luyando, P. Gunnellini, A. Harb, J. Hauk, M. Hempel, H. Jung, A. Kalogeropoulos, O. Karacheban, M. Kasemann, J. Keaveney, J. Kieseler, C. Kleinwort, I. Korol, W. Lange, A. Lelek, J. Leonard, K. Lipka, A. Lobanov, W. Lohmann, R. Mankel, I.-A. Melzer-Pellmann, A. B. Meyer, G. Mittag, J. Mnich, A. Mussgiller, E. Ntomari, D. Pitzl, R. Placakyte, A. Raspereza, B. Roland, M. Ö. Sahin, P. Saxena, T. Schoerner-Sadenius, C. Seitz, S. Spannagel, N. Stefaniuk, K. D. Trippkewitz, G. P. Van Onsem, R. Walsh, C. Wissing, V. Blobel, M. Centis Vignali, A. R. Draeger, T. Dreyer, E. Garutti, K. Goebel, D. Gonzalez, J. Haller, M. Hoffmann, R. S. Höing, A. Junkes, R. Klanner, R. Kogler, N. Kovalchuk, T. Lapsien, T. Lenz, I. Marchesini, D. Marconi, M. Meyer, M. Niedziela, D. Nowatschin, J. Ott, F. Pantaleo, T. Peiffer, A. Perieanu, J. Poehlsen, C. Sander, C. Scharf, P. Schleper, E. Schlieckau, A. Schmidt, S. Schumann, J. Schwandt, H. Stadie, G. Steinbrück, F. M. Stober, M. Stöver, H. Tholen, D. Troendle, E. Usai, L. Vanelderen, A. Vanhoefer, B. Vormwald, C. Barth, C. Baus, J. Berger, E. Butz, T. Chwalek, F. Colombo, W. De Boer, A. Dierlamm, S. Fink, R. Friese, M. Giffels, A. Gilbert, D. Haitz, F. Hartmann, S. M. Heindl, U. Husemann, I. Katkov, A. Kornmayer, P. Lobelle Pardo, B. Maier, H. Mildner, M. U. Mozer, T. Müller, Th. Müller, M. Plagge, G. Quast, K. Rabbertz, S. Röcker, F. Roscher, M. Schröder, G. Sieber, H. J. Simonis, R. Ulrich, J. Wagner-Kuhr, S. Wayand, M. Weber, T. Weiler, S. Williamson, C. Wöhrmann, R. Wolf, G. Anagnostou, G. Daskalakis, T. Geralis, V. A. Giakoumopoulou, A. Kyriakis, D. Loukas, I. Topsis-Giotis, A. Agapitos, S. Kesisoglou, A. Panagiotou, N. Saoulidou, E. Tziaferi, I. Evangelou, G. Flouris, C. Foudas, P. Kokkas, N. Loukas, N. Manthos, I. Papadopoulos, E. Paradas, N. Filipovic, G. Bencze, C. Hajdu, P. Hidas, D. Horvath, F. Sikler, V. Veszpremi, G. Vesztergombi, A. J. Zsigmond, N. Beni, S. Czellar, J. Karancsi, J. Molnar, Z. Szillasi, M. Bartók, A. Makovec, P. Raics, Z. L. Trocsanyi, B. Ujvari, S. Bahinipati, S. Choudhury, P. Mal, K. Mandal, A. Nayak, D. K. Sahoo, N. Sahoo, S. K. Swain, S. Bansal, S. B. Beri, V. Bhatnagar, R. Chawla, R. Gupta, U. Bhawandeep, A. K. Kalsi, A. Kaur, M. Kaur, R. Kumar, A. Mehta, M. Mittal, J. B. Singh, G. Walia, Ashok Kumar, A. Bhardwaj, B. C. Choudhary, R. B. Garg, S. Keshri, A. Kumar, S. Malhotra, M. Naimuddin, N. Nishu, K. Ranjan, R. Sharma, V. Sharma, R. Bhattacharya, S. Bhattacharya, K. Chatterjee, S. Dey, S. Dutt, S. Dutta, S. Ghosh, N. Majumdar, A. Modak, K. Mondal, S. Mukhopadhyay, S. Nandan, A. Purohit, A. Roy, D. Roy, S. Roy Chowdhury, S. Sarkar, M. Sharan, S. Thakur, P. K. Behera, R. Chudasama, D. Dutta, V. Jha, V. Kumar, A. K. Mohanty, P. K. Netrakanti, L. M. Pant, P. Shukla, A. Topkar, S. Bhowmik, R. K. Dewanjee, S. Ganguly, S. Kumar, M. Maity, B. Parida, T. Sarkar, T. Aziz, S. Dugad, G. Kole, B. Mahakud, S. Mitra, G. B. Mohanty, N. Sur, B. Sutar, S. Banerjee, M. Guchait, Sa. Jain, G. Majumder, K. Mazumdar, N. Wickramage, S. Chauhan, S. Dube, A. Kapoor, K. Kothekar, A. Rane, S. Sharma, H. Bakhshiansohi, H. Behnamian, S. Chenarani, E. Eskandari Tadavani, S. M. Etesami, A. Fahim, M. Khakzad, M. Mohammadi Najafabadi, M. Naseri, S. Paktinat Mehdiabadi, F. Rezaei Hosseinabadi, B. Safarzadeh, M. Zeinali, M. Felcini, M. Grunewald, M. Abbrescia, C. Calabria, C. Caputo, A. Colaleo, D. Creanza, L. Cristella, N. De Filippis, M. De Palma, L. Fiore, G. Iaselli, G. Maggi, M. Maggi, G. Miniello, S. My, S. Nuzzo, A. Pompili, G. Pugliese, R. Radogna, A. Ranieri, G. Selvaggi, L. Silvestris, R. Venditti, G. Abbiendi, C. Battilana, D. Bonacorsi, S. Braibant-Giacomelli, L. Brigliadori, R. Campanini, P. Capiluppi, A. Castro, F. R. Cavallo, S. S. Chhibra, G. Codispoti, M. Cuffiani, G. M. Dallavalle, F. Fabbri, A. Fanfani, D. Fasanella, P. Giacomelli, C. Grandi, L. Guiducci, S. Marcellini, G. Masetti, A. Montanari, F. L. Navarria, A. Perrotta, A. M. Rossi, T. Rovelli, G. P. Siroli, N. Tosi, S. Albergo, M. Chiorboli, S. Costa, A. Di Mattia, F. Giordano, R. Potenza, A. Tricomi, C. Tuve, G. Barbagli, V. Ciulli, C. Civinini, R. D’Alessandro, E. Focardi, V. Gori, P. Lenzi, M. Meschini, S. Paoletti, G. Sguazzoni, L. Viliani, L. Benussi, S. Bianco, F. Fabbri, D. Piccolo, F. Primavera, V. Calvelli, F. Ferro, M. Lo Vetere, M. R. Monge, E. Robutti, S. Tosi, L. Brianza, M. E. Dinardo, S. Fiorendi, S. Gennai, A. Ghezzi, P. Govoni, S. Malvezzi, R. A. Manzoni, B. Marzocchi, D. Menasce, L. Moroni, M. Paganoni, D. Pedrini, S. Pigazzini, S. Ragazzi, T. Tabarelli de Fatis, S. Buontempo, N. Cavallo, G. De Nardo, S. Di Guida, M. Esposito, F. Fabozzi, A. O. M. Iorio, G. Lanza, L. Lista, S. Meola, M. Merola, P. Paolucci, C. Sciacca, F. Thyssen, P. Azzi, N. Bacchetta, M. Bellato, L. Benato, D. Bisello, A. Boletti, R. Carlin, A. Carvalho Antunes De Oliveira, P. Checchia, M. Dall’Osso, P. De Castro Manzano, T. Dorigo, U. Gasparini, S. Lacaprara, M. Margoni, A. T. Meneguzzo, F. Montecassiano, M. Passaseo, J. Pazzini, N. Pozzobon, P. Ronchese, F. Simonetto, E. Torassa, S. Ventura, M. Zanetti, P. Zotto, A. Zucchetta, A. Braghieri, A. Magnani, P. Montagna, S. P. Ratti, V. Re, C. Riccardi, P. Salvini, I. Vai, P. Vitulo, L. Alunni Solestizi, G. M. Bilei, D. Ciangottini, L. Fanò, P. Lariccia, R. Leonardi, G. Mantovani, M. Menichelli, A. Saha, A. Santocchia, K. Androsov, P. Azzurri, G. Bagliesi, J. Bernardini, T. Boccali, R. Castaldi, M. A. Ciocci, R. Dell’Orso, S. Donato, G. Fedi, A. Giassi, M. T. Grippo, F. Ligabue, T. Lomtadze, L. Martini, A. Messineo, F. Palla, A. Rizzi, A. Savoy-Navarro, P. Spagnolo, R. Tenchini, G. Tonelli, A. Venturi, P. G. Verdini, L. Barone, F. Cavallari, M. Cipriani, G. D’imperio, D. Del Re, M. Diemoz, S. Gelli, C. Jorda, E. Longo, F. Margaroli, P. Meridiani, G. Organtini, R. Paramatti, F. Preiato, S. Rahatlou, C. Rovelli, F. Santanastasio, N. Amapane, R. Arcidiacono, S. Argiro, M. Arneodo, N. Bartosik, R. Bellan, C. Biino, N. Cartiglia, M. Costa, R. Covarelli, A. Degano, N. Demaria, L. Finco, B. Kiani, C. Mariotti, S. Maselli, E. Migliore, V. Monaco, E. Monteil, M. M. Obertino, L. Pacher, N. Pastrone, M. Pelliccioni, G. L. Pinna Angioni, F. Ravera, A. Romero, M. Ruspa, R. Sacchi, K. Shchelina, V. Sola, A. Solano, A. Staiano, P. Traczyk, S. Belforte, M. Casarsa, F. Cossutti, G. Della Ricca, C. La Licata, A. Schizzi, A. Zanetti, D. H. Kim, G. N. Kim, M. S. Kim, S. Lee, S. W. Lee, Y. D. Oh, S. Sekmen, D. C. Son, Y. C. Yang, H. Kim, A. Lee, J. A. Brochero Cifuentes, T. J. Kim, S. Cho, S. Choi, Y. Go, D. Gyun, S. Ha, B. Hong, Y. Jo, Y. Kim, B. Lee, K. Lee, K. S. Lee, S. Lee, J. Lim, S. K. Park, Y. Roh, J. Almond, J. Kim, S. B. Oh, S. h. Seo, U. K. Yang, H. D. Yoo, G. B. Yu, M. Choi, H. Kim, H. Kim, J. H. Kim, J. S. H. Lee, I. C. Park, G. Ryu, M. S. Ryu, Y. Choi, J. Goh, D. Kim, E. Kwon, J. Lee, I. Yu, V. Dudenas, A. Juodagalvis, J. Vaitkus, I. Ahmed, Z. A. Ibrahim, J. R. Komaragiri, M. A. B. Md Ali, F. Mohamad Idris, W. A. T. Wan Abdullah, M. N. Yusli, Z. Zolkapli, H. Castilla-Valdez, E. De La Cruz-Burelo, I. Heredia-De La Cruz, A. Hernandez-Almada, R. Lopez-Fernandez, J. Mejia Guisao, A. Sanchez-Hernandez, S. Carrillo Moreno, C. Oropeza Barrera, F. Vazquez Valencia, S. Carpinteyro, I. Pedraza, H. A. Salazar Ibarguen, C. Uribe Estrada, A. Morelos Pineda, D. Krofcheck, P. H. Butler, A. Ahmad, M. Ahmad, Q. Hassan, H. R. Hoorani, W. A. Khan, M A. Shah, M. Shoaib, M. Waqas, H. Bialkowska, M. Bluj, B. Boimska, T. Frueboes, M. Górski, M. Kazana, K. Nawrocki, K. Romanowska-Rybinska, M. Szleper, P. Zalewski, K. Bunkowski, A. Byszuk, K. Doroba, A. Kalinowski, M. Konecki, J. Krolikowski, M. Misiura, M. Olszewski, M. Walczak, P. Bargassa, C. Beirão Da Cruz E Silva, A. Di Francesco, P. Faccioli, P. G. Ferreira Parracho, M. Gallinaro, J. Hollar, N. Leonardo, L. Lloret Iglesias, M. V. Nemallapudi, J. Rodrigues Antunes, J. Seixas, O. Toldaiev, D. Vadruccio, J. Varela, P. Vischia, S. Afanasiev, P. Bunin, M. Gavrilenko, I. Golutvin, I. Gorbunov, A. Kamenev, V. Karjavin, A. Lanev, A. Malakhov, V. Matveev, P. Moisenz, V. Palichik, V. Perelygin, S. Shmatov, S. Shulha, N. Skatchkov, V. Smirnov, N. Voytishin, A. Zarubin, L. Chtchipounov, V. Golovtsov, Y. Ivanov, V. Kim, E. Kuznetsova, V. Murzin, V. Oreshkin, V. Sulimov, A. Vorobyev, Yu. Andreev, A. Dermenev, S. Gninenko, N. Golubev, A. Karneyeu, M. Kirsanov, N. Krasnikov, A. Pashenkov, D. Tlisov, A. Toropin, V. Epshteyn, V. Gavrilov, N. Lychkovskaya, V. Popov, l. Pozdnyakov, G. Safronov, A. Spiridonov, M. Toms, E. Vlasov, A. Zhokin, M. Chadeeva, M. Danilov, O. Markin, V. Andreev, M. Azarkin, I. Dremin, M. Kirakosyan, A. Leonidov, S. V. Rusakov, A. Terkulov, A. Baskakov, A. Belyaev, E. Boos, M. Dubinin, L. Dudko, A. Ershov, A. Gribushin, V. Klyukhin, O. Kodolova, I. Lokhtin, I. Miagkov, S. Obraztsov, S. Petrushanko, V. Savrin, A Snigirev, I. Azhgirey, I. Bayshev, S. Bitioukov, D. Elumakhov, V. Kachanov, A. Kalinin, D. Konstantinov, V. Krychkine, V. Petrov, R. Ryutin, A. Sobol, S. Troshin, N. Tyurin, A. Uzunian, A. Volkov, P. Adzic, P. Cirkovic, D. Devetak, J. Milosevic, V. Rekovic, J. Alcaraz Maestre, E. Calvo, M. Cerrada, M. Chamizo Llatas, N. Colino, B. De La Cruz, A. Delgado Peris, A. Escalante Del Valle, C. Fernandez Bedoya, J. P. Fernández Ramos, J. Flix, M. C. Fouz, P. Garcia-Abia, O. Gonzalez Lopez, S. Goy Lopez, J. M. Hernandez, M. I. Josa, E. Navarro De Martino, A. Pérez-Calero Yzquierdo, J. Puerta Pelayo, A. Quintario Olmeda, I. Redondo, L. Romero, M. S. Soares, J. F. de Trocóniz, M. Missiroli, D. Moran, J. Cuevas, J. Fernandez Menendez, I. Gonzalez Caballero, J. R. González Fernández, E. Palencia Cortezon, S. Sanchez Cruz, J. M. Vizan Garcia, I. J. Cabrillo, A. Calderon, J. R. Castiñeiras De Saa, E. Curras, M. Fernandez, J. Garcia-Ferrero, G. Gomez, A. Lopez Virto, J. Marco, C. Martinez Rivero, F. Matorras, J. Piedra Gomez, T. Rodrigo, A. Ruiz-Jimeno, L. Scodellaro, N. Trevisani, I. Vila, R. Vilar Cortabitarte, D. Abbaneo, E. Auffray, G. Auzinger, M. Bachtis, P. Baillon, A. H. Ball, D. Barney, P. Bloch, A. Bocci, A. Bonato, C. Botta, T. Camporesi, R. Castello, M. Cepeda, G. Cerminara, M. D’Alfonso, D. d’Enterria, A. Dabrowski, V. Daponte, A. David, M. De Gruttola, F. De Guio, A. De Roeck, E. Di Marco, M. Dobson, M. Dordevic, B. Dorney, T. du Pree, D. Duggan, M. Dünser, N. Dupont, A. Elliott-Peisert, S. Fartoukh, G. Franzoni, J. Fulcher, W. Funk, D. Gigi, K. Gill, M. Girone, F. Glege, D. Gulhan, S. Gundacker, M. Guthoff, J. Hammer, P. Harris, J. Hegeman, V. Innocente, P. Janot, H. Kirschenmann, V. Knünz, M. J. Kortelainen, K. Kousouris, M. Krammer, P. Lecoq, C. Lourenço, M. T. Lucchini, L. Malgeri, M. Mannelli, A. Martelli, F. Meijers, S. Mersi, E. Meschi, F. Moortgat, S. Morovic, M. Mulders, H. Neugebauer, S. Orfanelli, L. Orsini, L. Pape, E. Perez, M. Peruzzi, A. Petrilli, G. Petrucciani, A. Pfeiffer, M. Pierini, A. Racz, T. Reis, G. Rolandi, M. Rovere, M. Ruan, H. Sakulin, J. B. Sauvan, C. Schäfer, C. Schwick, M. Seidel, A. Sharma, P. Silva, M. Simon, P. Sphicas, J. Steggemann, M. Stoye, Y. Takahashi, M. Tosi, D. Treille, A. Triossi, A. Tsirou, V. Veckalns, G. I. Veres, N. Wardle, A. Zagozdzinska, W. D. Zeuner, W. Bertl, K. Deiters, W. Erdmann, R. Horisberger, Q. Ingram, H. C. Kaestli, D. Kotlinski, U. Langenegger, T. Rohe, F. Bachmair, L. Bäni, L. Bianchini, B. Casal, G. Dissertori, M. Dittmar, M. Donegà, P. Eller, C. Grab, C. Heidegger, D. Hits, J. Hoss, G. Kasieczka, P. Lecomte, W. Lustermann, B. Mangano, M. Marionneau, P. Martinez Ruiz del Arbol, M. Masciovecchio, M. T. Meinhard, D. Meister, F. Micheli, P. Musella, F. Nessi-Tedaldi, F. Pandolfi, J. Pata, F. Pauss, G. Perrin, L. Perrozzi, M. Quittnat, M. Rossini, M. Schönenberger, A. Starodumov, M. Takahashi, V. R. Tavolaro, K. Theofilatos, R. Wallny, T. K. Aarrestad, C. Amsler, L. Caminada, M. F. Canelli, V. Chiochia, A. De Cosa, C. Galloni, A. Hinzmann, T. Hreus, B. Kilminster, C. Lange, J. Ngadiuba, D. Pinna, G. Rauco, P. Robmann, D. Salerno, Y. Yang, V. Candelise, T. H. Doan, Sh. Jain, R. Khurana, M. Konyushikhin, C. M. Kuo, W. Lin, Y. J. Lu, A. Pozdnyakov, S. S. Yu, Arun Kumar, P. Chang, Y. H. Chang, Y. W. Chang, Y. Chao, K. F. Chen, P. H. Chen, C. Dietz, F. Fiori, W.-S. Hou, Y. Hsiung, Y. F. Liu, R.-S. Lu, M. Miñano Moya, E. Paganis, A. Psallidas, J. F. Tsai, Y. M. Tzeng, B. Asavapibhop, G. Singh, N. Srimanobhas, N. Suwonjandee, A. Adiguzel, S. Cerci, S. Damarseckin, Z. S. Demiroglu, C. Dozen, I. Dumanoglu, S. Girgis, G. Gokbulut, Y. Guler, E. Gurpinar, I. Hos, E. E. Kangal, G. Onengut, K. Ozdemir, D. Sunar Cerci, B. Tali, H. Topakli, S. Turkcapar, C. Zorbilmez, B. Bilin, S. Bilmis, B. Isildak, G. Karapinar, M. Yalvac, M. Zeyrek, E. Gülmez, M. Kaya, O. Kaya, E. A. Yetkin, T. Yetkin, A. Cakir, K. Cankocak, S. Sen, B. Grynyov, L. Levchuk, P. Sorokin, R. Aggleton, F. Ball, L. Beck, J. J. Brooke, D. Burns, E. Clement, D. Cussans, H. Flacher, J. Goldstein, M. Grimes, G. P. Heath, H. F. Heath, J. Jacob, L. Kreczko, C. Lucas, D. M. Newbold, S. Paramesvaran, A. Poll, T. Sakuma, S. Seif El Nasr-Storey, D. Smith, V. J. Smith, K. W. Bell, A. Belyaev, C. Brew, R. M. Brown, L. Calligaris, D. Cieri, D. J. A. Cockerill, J. A. Coughlan, K. Harder, S. Harper, E. Olaiya, D. Petyt, C. H. Shepherd-Themistocleous, A. Thea, I. R. Tomalin, T. Williams, M. Baber, R. Bainbridge, O. Buchmuller, A. Bundock, D. Burton, S. Casasso, M. Citron, D. Colling, L. Corpe, P. Dauncey, G. Davies, A. De Wit, M. Della Negra, P. Dunne, A. Elwood, D. Futyan, Y. Haddad, G. Hall, G. Iles, R. Lane, C. Laner, R. Lucas, L. Lyons, A.-M. Magnan, S. Malik, L. Mastrolorenzo, J. Nash, A. Nikitenko, J. Pela, B. Penning, M. Pesaresi, D. M. Raymond, A. Richards, A. Rose, C. Seez, A. Tapper, K. Uchida, M. Vazquez Acosta, T. Virdee, S. C. Zenz, J. E. Cole, P. R. Hobson, A. Khan, P. Kyberd, D. Leslie, I. D. Reid, P. Symonds, L. Teodorescu, M. Turner, A. Borzou, K. Call, J. Dittmann, K. Hatakeyama, H. Liu, N. Pastika, O. Charaf, S. I. Cooper, C. Henderson, P. Rumerio, D. Arcaro, A. Avetisyan, T. Bose, D. Gastler, D. Rankin, C. Richardson, J. Rohlf, L. Sulak, D. Zou, G. Benelli, E. Berry, D. Cutts, A. Ferapontov, A. Garabedian, J. Hakala, U. Heintz, O. Jesus, E. Laird, G. Landsberg, Z. Mao, M. Narain, S. Piperov, S. Sagir, E. Spencer, R. Syarif, R. Breedon, G. Breto, D. Burns, M. Calderon De La Barca Sanchez, S. Chauhan, M. Chertok, J. Conway, R. Conway, P. T. Cox, R. Erbacher, C. Flores, G. Funk, M. Gardner, W. Ko, R. Lander, C. Mclean, M. Mulhearn, D. Pellett, J. Pilot, F. Ricci-Tam, S. Shalhout, J. Smith, M. Squires, D. Stolp, M. Tripathi, S. Wilbur, R. Yohay, R. Cousins, P. Everaerts, A. Florent, J. Hauser, M. Ignatenko, D. Saltzberg, E. Takasugi, V. Valuev, M. Weber, K. Burt, R. Clare, J. Ellison, J. W. Gary, G. Hanson, J. Heilman, P. Jandir, E. Kennedy, F. Lacroix, O. R. Long, M. Malberti, M. Olmedo Negrete, M. I. Paneva, A. Shrinivas, H. Wei, S. Wimpenny, B. R. Yates, J. G. Branson, G. B. Cerati, S. Cittolin, M. Derdzinski, R. Gerosa, A. Holzner, D. Klein, J. Letts, I. Macneill, D. Olivito, S. Padhi, M. Pieri, M. Sani, V. Sharma, S. Simon, M. Tadel, A. Vartak, S. Wasserbaech, C. Welke, J. Wood, F. Würthwein, A. Yagil, G. Zevi Della Porta, R. Bhandari, J. Bradmiller-Feld, C. Campagnari, A. Dishaw, V. Dutta, K. Flowers, M. Franco Sevilla, P. Geffert, C. George, F. Golf, L. Gouskos, J. Gran, R. Heller, J. Incandela, N. Mccoll, S. D. Mullin, A. Ovcharova, J. Richman, D. Stuart, I. Suarez, C. West, J. Yoo, D. Anderson, A. Apresyan, J. Bendavid, A. Bornheim, J. Bunn, Y. Chen, J. Duarte, A. Mott, H. B. Newman, C. Pena, M. Spiropulu, J. R. Vlimant, S. Xie, R. Y. Zhu, M. B. Andrews, V. Azzolini, A. Calamba, B. Carlson, T. Ferguson, M. Paulini, J. Russ, M. Sun, H. Vogel, I. Vorobiev, J. P. Cumalat, W. T. Ford, F. Jensen, A. Johnson, M. Krohn, T. Mulholland, K. Stenson, S. R. Wagner, J. Alexander, J. Chaves, J. Chu, S. Dittmer, N. Mirman, G. Nicolas Kaufman, J. R. Patterson, A. Rinkevicius, A. Ryd, L. Skinnari, W. Sun, S. M. Tan, Z. Tao, J. Thom, J. Tucker, P. Wittich, D. Winn, S. Abdullin, M. Albrow, G. Apollinari, S. Banerjee, L. A. T. Bauerdick, A. Beretvas, J. Berryhill, P. C. Bhat, G. Bolla, K. Burkett, J. N. Butler, H. W. K. Cheung, F. Chlebana, S. Cihangir, M. Cremonesi, V. D. Elvira, I. Fisk, J. Freeman, E. Gottschalk, L. Gray, D. Green, S. Grünendahl, O. Gutsche, D. Hare, R. M. Harris, S. Hasegawa, J. Hirschauer, Z. Hu, B. Jayatilaka, S. Jindariani, M. Johnson, U. Joshi, B. Klima, B. Kreis, S. Lammel, J. Linacre, D. Lincoln, R. Lipton, T. Liu, R. Lopes De Sá, J. Lykken, K. Maeshima, N. Magini, J. M. Marraffino, S. Maruyama, D. Mason, P. McBride, P. Merkel, S. Mrenna, S. Nahn, C. Newman-Holmes, V. O’Dell, K. Pedro, O. Prokofyev, G. Rakness, L. Ristori, E. Sexton-Kennedy, A. Soha, W. J. Spalding, L. Spiegel, S. Stoynev, N. Strobbe, L. Taylor, S. Tkaczyk, N. V. Tran, L. Uplegger, E. W. Vaandering, C. Vernieri, M. Verzocchi, R. Vidal, M. Wang, H. A. Weber, A. Whitbeck, D. Acosta, P. Avery, P. Bortignon, D. Bourilkov, A. Brinkerhoff, A. Carnes, M. Carver, D. Curry, S. Das, R. D. Field, I. K. Furic, J. Konigsberg, A. Korytov, P. Ma, K. Matchev, H. Mei, P. Milenovic, G. Mitselmakher, D. Rank, L. Shchutska, D. Sperka, L. Thomas, J. Wang, S. Wang, J. Yelton, S. Linn, P. Markowitz, G. Martinez, J. L. Rodriguez, A. Ackert, J. R. Adams, T. Adams, A. Askew, S. Bein, B. Diamond, S. Hagopian, V. Hagopian, K. F. Johnson, A. Khatiwada, H. Prosper, A. Santra, M. Weinberg, M. M. Baarmand, V. Bhopatkar, S. Colafranceschi, M. Hohlmann, D. Noonan, T. Roy, F. Yumiceva, M. R. Adams, L. Apanasevich, D. Berry, R. R. Betts, I. Bucinskaite, R. Cavanaugh, O. Evdokimov, L. Gauthier, C. E. Gerber, D. J. Hofman, P. Kurt, C. O’Brien, l. D. Sandoval Gonzalez, P. Turner, N. Varelas, Z. Wu, M. Zakaria, J. Zhang, B. Bilki, W. Clarida, K. Dilsiz, S. Durgut, R. P. Gandrajula, M. Haytmyradov, V. Khristenko, J.-P. Merlo, H. Mermerkaya, A. Mestvirishvili, A. Moeller, J. Nachtman, H. Ogul, Y. Onel, F. Ozok, A. Penzo, C. Snyder, E. Tiras, J. Wetzel, K. Yi, I. Anderson, B. Blumenfeld, A. Cocoros, N. Eminizer, D. Fehling, L. Feng, A. V. Gritsan, P. Maksimovic, M. Osherson, J. Roskes, U. Sarica, M. Swartz, M. Xiao, Y. Xin, C. You, A. Al-bataineh, P. Baringer, A. Bean, J. Bowen, C. Bruner, J. Castle, R. P. Kenny, A. Kropivnitskaya, D. Majumder, W. Mcbrayer, M. Murray, S. Sanders, R. Stringer, J. D. Tapia Takaki, Q. Wang, A. Ivanov, K. Kaadze, S. Khalil, M. Makouski, Y. Maravin, A. Mohammadi, L. K. Saini, N. Skhirtladze, S. Toda, D. Lange, F. Rebassoo, D. Wright, C. Anelli, A. Baden, O. Baron, A. Belloni, B. Calvert, S. C. Eno, C. Ferraioli, J. A. Gomez, N. J. Hadley, S. Jabeen, R. G. Kellogg, T. Kolberg, J. Kunkle, Y. Lu, A. C. Mignerey, Y. H. Shin, A. Skuja, M. B. Tonjes, S. C. Tonwar, A. Apyan, R. Barbieri, A. Baty, R. Bi, K. Bierwagen, S. Brandt, W. Busza, I. A. Cali, Z. Demiragli, L. Di Matteo, G. Gomez Ceballos, M. Goncharov, D. Hsu, Y. Iiyama, G. M. Innocenti, M. Klute, D. Kovalskyi, K. Krajczar, Y. S. Lai, Y.-J. Lee, A. Levin, P. D. Luckey, A. C. Marini, C. Mcginn, C. Mironov, S. Narayanan, X. Niu, C. Paus, C. Roland, G. Roland, J. Salfeld-Nebgen, G. S. F. Stephans, K. Sumorok, K. Tatar, M. Varma, D. Velicanu, J. Veverka, J. Wang, T. W. Wang, B. Wyslouch, M. Yang, V. Zhukova, A. C. Benvenuti, R. M. Chatterjee, A. Evans, A. Finkel, A. Gude, P. Hansen, S. Kalafut, S. C. Kao, Y. Kubota, Z. Lesko, J. Mans, S. Nourbakhsh, N. Ruckstuhl, R. Rusack, N. Tambe, J. Turkewitz, J. G. Acosta, S. Oliveros, E. Avdeeva, R. Bartek, K. Bloom, S. Bose, D. R. Claes, A. Dominguez, C. Fangmeier, R. Gonzalez Suarez, R. Kamalieddin, D. Knowlton, I. Kravchenko, A. Malta Rodrigues, F. Meier, J. Monroy, J. E. Siado, G. R. Snow, B. Stieger, M. Alyari, J. Dolen, J. George, A. Godshalk, C. Harrington, I. Iashvili, J. Kaisen, A. Kharchilava, A. Kumar, A. Parker, S. Rappoccio, B. Roozbahani, G. Alverson, E. Barberis, D. Baumgartel, M. Chasco, A. Hortiangtham, A. Massironi, D. M. Morse, D. Nash, T. Orimoto, R. Teixeira De Lima, D. Trocino, R.-J. Wang, D. Wood, S. Bhattacharya, K. A. Hahn, A. Kubik, J. F. Low, N. Mucia, N. Odell, B. Pollack, M. H. Schmitt, K. Sung, M. Trovato, M. Velasco, N. Dev, M. Hildreth, K. Hurtado Anampa, C. Jessop, D. J. Karmgard, N. Kellams, K. Lannon, N. Marinelli, F. Meng, C. Mueller, Y. Musienko, M. Planer, A. Reinsvold, R. Ruchti, G. Smith, S. Taroni, N. Valls, M. Wayne, M. Wolf, A. Woodard, J. Alimena, L. Antonelli, J. Brinson, B. Bylsma, L. S. Durkin, S. Flowers, B. Francis, A. Hart, C. Hill, R. Hughes, W. Ji, B. Liu, W. Luo, D. Puigh, B. L. Winer, H. W. Wulsin, S. Cooperstein, O. Driga, P. Elmer, J. Hardenbrook, P. Hebda, J. Luo, D. Marlow, T. Medvedeva, M. Mooney, J. Olsen, C. Palmer, P. Piroué, D. Stickland, C. Tully, A. Zuranski, S. Malik, A. Barker, V. E. Barnes, D. Benedetti, S. Folgueras, L. Gutay, M. K. Jha, M. Jones, A. W. Jung, K. Jung, D. H. Miller, N. Neumeister, B. C. Radburn-Smith, X. Shi, J. Sun, A. Svyatkovskiy, F. Wang, W. Xie, L. Xu, N. Parashar, J. Stupak, A. Adair, B. Akgun, Z. Chen, K. M. Ecklund, F. J. M. Geurts, M. Guilbaud, W. Li, B. Michlin, M. Northup, B. P. Padley, R. Redjimi, J. Roberts, J. Rorie, Z. Tu, J. Zabel, B. Betchart, A. Bodek, P. de Barbaro, R. Demina, Y. t. Duh, T. Ferbel, M. Galanti, A. Garcia-Bellido, J. Han, O. Hindrichs, A. Khukhunaishvili, K. H. Lo, P. Tan, M. Verzetti, J. P. Chou, E. Contreras-Campana, Y. Gershtein, T. A. Gómez Espinosa, E. Halkiadakis, M. Heindl, D. Hidas, E. Hughes, S. Kaplan, R. Kunnawalkam Elayavalli, S. Kyriacou, A. Lath, K. Nash, H. Saka, S. Salur, S. Schnetzer, D. Sheffield, S. Somalwar, R. Stone, S. Thomas, P. Thomassen, M. Walker, M. Foerster, J. Heideman, G. Riley, K. Rose, S. Spanier, K. Thapa, O. Bouhali, A. Castaneda Hernandez, A. Celik, M. Dalchenko, M. De Mattia, A. Delgado, S. Dildick, R. Eusebi, J. Gilmore, T. Huang, E. Juska, T. Kamon, V. Krutelyov, R. Mueller, Y. Pakhotin, R. Patel, A. Perloff, L. Perniè, D. Rathjens, A. Rose, A. Safonov, A. Tatarinov, K. A. Ulmer, N. Akchurin, C. Cowden, J. Damgov, C. Dragoiu, P. R. Dudero, J. Faulkner, S. Kunori, K. Lamichhane, S. W. Lee, T. Libeiro, S. Undleeb, I. Volobouev, Z. Wang, A. G. Delannoy, S. Greene, A. Gurrola, R. Janjam, W. Johns, C. Maguire, A. Melo, H. Ni, P. Sheldon, S. Tuo, J. Velkovska, Q. Xu, M. W. Arenton, P. Barria, B. Cox, J. Goodell, R. Hirosky, A. Ledovskoy, H. Li, C. Neu, T. Sinthuprasith, X. Sun, Y. Wang, E. Wolfe, F. Xia, C. Clarke, R. Harr, P. E. Karchin, P. Lamichhane, J. Sturdy, D. A. Belknap, S. Dasu, L. Dodd, S. Duric, B. Gomber, M. Grothe, M. Herndon, A. Hervé, P. Klabbers, A. Lanaro, A. Levine, K. Long, R. Loveless, I. Ojalvo, T. Perry, G. A. Pierro, G. Polese, T. Ruggles, A. Savin, A. Sharma, N. Smith, W. H. Smith, D. Taylor, P. Verwilligen, N. Woods, [Authorinst]The CMS Collaboration

**Affiliations:** 1 Yerevan Physics Institute, Yerevan, Armenia; 2 Institut für Hochenergiephysik der OeAW, Vienna, Austria; 3 National Centre for Particle and High Energy Physics, Minsk, Belarus; 4 Universiteit Antwerpen, Antwerp, Belgium; 5 Vrije Universiteit Brussel, Brussels, Belgium; 6 Université Libre de Bruxelles, Brussels, Belgium; 7 Ghent University, Ghent, Belgium; 8 Université Catholique de Louvain, Louvain-la-Neuve, Belgium; 9 Université de Mons, Mons, Belgium; 10 Centro Brasileiro de Pesquisas Fisicas, Rio de Janeiro, Brazil; 11 Universidade do Estado do Rio de Janeiro, Rio de Janeiro, Brazil; 12 Universidade Estadual Paulista, Universidade Federal do ABC, São Paulo, Brazil; 13 Institute for Nuclear Research and Nuclear Energy, Sofia, Bulgaria; 14 University of Sofia, Sofia, Bulgaria; 15 Beihang University, Beijing, China; 16 Institute of High Energy Physics, Beijing, China; 17 State Key Laboratory of Nuclear Physics and Technology, Peking University, Beijing, China; 18 Universidad de Los Andes, Bogotá, Colombia; 19 Faculty of Electrical Engineering, Mechanical Engineering and Naval Architecture, University of Split, Split, Croatia; 20 Faculty of Science, University of Split, Split, Croatia; 21 Institute Rudjer Boskovic, Zagreb, Croatia; 22 University of Cyprus, Nicosia, Cyprus; 23 Charles University, Prague, Czech Republic; 24 Universidad San Francisco de Quito, Quito, Ecuador; 25 Academy of Scientific Research and Technology of the Arab Republic of Egypt, Egyptian Network of High Energy Physics, Cairo, Egypt; 26 National Institute of Chemical Physics and Biophysics, Tallinn, Estonia; 27 Department of Physics, University of Helsinki, Helsinki, Finland; 28 Helsinki Institute of Physics, Helsinki, Finland; 29 Lappeenranta University of Technology, Lappeenranta, Finland; 30 DSM/IRFU, CEA/Saclay, Gif-sur-Yvette, France; 31 Laboratoire Leprince-Ringuet, Ecole Polytechnique, IN2P3-CNRS, Palaiseau, France; 32 Institut Pluridisciplinaire Hubert Curien, Université de Strasbourg, Université de Haute Alsace Mulhouse, CNRS/IN2P3, Strasbourg, France; 33 Centre de Calcul de l’Institut National de Physique Nucleaire et de Physique des Particules, CNRS/IN2P3, Villeurbanne, France; 34 Institut de Physique Nucléaire de Lyon, Université de Lyon, Université Claude Bernard Lyon 1, CNRS-IN2P3, Villeurbanne, France; 35 Georgian Technical University, Tbilisi, Georgia; 36 Tbilisi State University, Tbilisi, Georgia; 37 I. Physikalisches Institut, RWTH Aachen University, Aachen, Germany; 38 III. Physikalisches Institut A, RWTH Aachen University, Aachen, Germany; 39 III. Physikalisches Institut B, RWTH Aachen University, Aachen, Germany; 40 Deutsches Elektronen-Synchrotron, Hamburg, Germany; 41 University of Hamburg, Hamburg, Germany; 42 Institut für Experimentelle Kernphysik, Karlsruhe, Germany; 43 Institute of Nuclear and Particle Physics (INPP), NCSR Demokritos, Aghia Paraskevi, Greece; 44 National and Kapodistrian University of Athens, Athens, Greece; 45 University of Ioánnina, Ioannina, Greece; 46 MTA-ELTE Lendület CMS Particle and Nuclear Physics Group, Eötvös Loránd University, Budapest, Hungary; 47 Wigner Research Centre for Physics, Budapest, Hungary; 48 Institute of Nuclear Research ATOMKI, Debrecen, Hungary; 49 University of Debrecen, Debrecen, Hungary; 50 National Institute of Science Education and Research, Bhubaneswar, India; 51 Panjab University, Chandigarh, India; 52 University of Delhi, Delhi, India; 53 Saha Institute of Nuclear Physics, Kolkata, India; 54 Indian Institute of Technology Madras, Madras, India; 55 Bhabha Atomic Research Centre, Mumbai, India; 56 Tata Institute of Fundamental Research, Mumbai, India; 57 Tata Institute of Fundamental Research-A, Mumbai, India; 58 Tata Institute of Fundamental Research-B, Mumbai, India; 59 Indian Institute of Science Education and Research (IISER), Pune, India; 60 Institute for Research in Fundamental Sciences (IPM), Tehran, Iran; 61 University College Dublin, Dublin, Ireland; 62 INFN Sezione di Bari, Università di Bari, Politecnico di Bari, Bari, Italy; 63 INFN Sezione di Bologna, Università di Bologna, Bologna, Italy; 64 INFN Sezione di Catania, Università di Catania, Catania, Italy; 65 INFN Sezione di Firenze, Università di Firenze, Florence, Italy; 66 INFN Laboratori Nazionali di Frascati, Frascati, Italy; 67 INFN Sezione di Genova, Università di Genova, Genova, Italy; 68 INFN Sezione di Milano-Bicocca, Università di Milano-Bicocca, Milan, Italy; 69 INFN Sezione di Napoli, Università di Napoli ‘Federico II’, Napoli, Italy, Università della Basilicata, Potenza, Italy, Università G. Marconi, Rome, Italy; 70 INFN Sezione di Padova, Università di Padova, Padua, Italy, Università di Trento, Trento, Italy; 71 INFN Sezione di Pavia, Università di Pavia, Pavia, Italy; 72 INFN Sezione di Perugia, Università di Perugia, Perugia, Italy; 73 INFN Sezione di Pisa, Università di Pisa, Scuola Normale Superiore di Pisa, Pisa, Italy; 74 INFN Sezione di Roma, Università di Roma, Rome, Italy; 75 INFN Sezione di Torino, Università di Torino, Turin, Italy, Università del Piemonte Orientale, Novara, Italy; 76 INFN Sezione di Trieste, Università di Trieste, Trieste, Italy; 77 Kyungpook National University, Taegu, Korea; 78 Chonbuk National University, Jeonju, Korea; 79 Hanyang University, Seoul, Korea; 80 Korea University, Seoul, Korea; 81 Seoul National University, Seoul, Korea; 82 University of Seoul, Seoul, Korea; 83 Sungkyunkwan University, Suwon, Korea; 84 Vilnius University, Vilnius, Lithuania; 85 National Centre for Particle Physics, Universiti Malaya, Kuala Lumpur, Malaysia; 86 Centro de Investigacion y de Estudios Avanzados del IPN, Mexico City, Mexico; 87 Universidad Iberoamericana, Mexico City, Mexico; 88 Benemerita Universidad Autonoma de Puebla, Puebla, Mexico; 89 Universidad Autónoma de San Luis Potosí, San Luis Potosí, Mexico; 90 University of Auckland, Auckland, New Zealand; 91 University of Canterbury, Christchurch, New Zealand; 92 National Centre for Physics, Quaid-I-Azam University, Islamabad, Pakistan; 93 National Centre for Nuclear Research, Swierk, Poland; 94 Faculty of Physics, Institute of Experimental Physics, University of Warsaw, Warsaw, Poland; 95 Laboratório de Instrumentação e Física Experimental de Partículas, Lisbon, Portugal; 96 Joint Institute for Nuclear Research, Dubna, Russia; 97 Petersburg Nuclear Physics Institute, Gatchina (St. Petersburg), Russia; 98 Institute for Nuclear Research, Moscow, Russia; 99 Institute for Theoretical and Experimental Physics, Moscow, Russia; 100 National Research Nuclear University ‘Moscow Engineering Physics Institute’ (MEPhI), Moscow, Russia; 101 P. N. Lebedev Physical Institute, Moscow, Russia; 102 Skobeltsyn Institute of Nuclear Physics, Lomonosov Moscow State University, Moscow, Russia; 103 State Research Center of Russian Federation, Institute for High Energy Physics, Protvino, Russia; 104 Faculty of Physics and Vinca Institute of Nuclear Sciences, University of Belgrade, Belgrade, Serbia; 105 Centro de Investigaciones Energéticas Medioambientales y Tecnológicas (CIEMAT), Madrid, Spain; 106 Universidad Autónoma de Madrid, Madrid, Spain; 107 Universidad de Oviedo, Oviedo, Spain; 108 Instituto de Física de Cantabria (IFCA), CSIC-Universidad de Cantabria, Santander, Spain; 109 CERN, European Organization for Nuclear Research, Geneva, Switzerland; 110 Paul Scherrer Institut, Villigen, Switzerland; 111 Institute for Particle Physics, ETH Zurich, Zurich, Switzerland; 112 Universität Zürich, Zurich, Switzerland; 113 National Central University, Chung-Li, Taiwan; 114 National Taiwan University (NTU), Taipei, Taiwan; 115 Department of Physics, Faculty of Science, Chulalongkorn University, Bangkok, Thailand; 116 Cukurova University, Adana, Turkey; 117 Physics Department, Middle East Technical University, Ankara, Turkey; 118 Bogazici University, Istanbul, Turkey; 119 Istanbul Technical University, Istanbul, Turkey; 120 Institute for Scintillation Materials of National Academy of Science of Ukraine, Kharkiv, Ukraine; 121 National Scientific Center, Kharkov Institute of Physics and Technology, Kharkov, Ukraine; 122 University of Bristol, Bristol, UK; 123 Rutherford Appleton Laboratory, Didcot, UK; 124 Imperial College, London, UK; 125 Brunel University, Uxbridge, UK; 126 Baylor University, Waco, USA; 127 The University of Alabama, Tuscaloosa, USA; 128 Boston University, Boston, USA; 129 Brown University, Providence, USA; 130 University of California, Davis, Davis, USA; 131 University of California, Los Angeles, USA; 132 University of California, Riverside, Riverside, USA; 133 University of California, San Diego, La Jolla, USA; 134 University of California, Santa Barbara, Santa Barbara, USA; 135 California Institute of Technology, Pasadena, USA; 136 Carnegie Mellon University, Pittsburgh, USA; 137 University of Colorado Boulder, Boulder, USA; 138 Cornell University, Ithaca, USA; 139 Fairfield University, Fairfield, USA; 140 Fermi National Accelerator Laboratory, Batavia, USA; 141 University of Florida, Gainesville, USA; 142 Florida International University, Miami, USA; 143 Florida State University, Tallahassee, USA; 144 Florida Institute of Technology, Melbourne, USA; 145 University of Illinois at Chicago (UIC), Chicago, USA; 146 The University of Iowa, Iowa City, USA; 147 Johns Hopkins University, Baltimore, USA; 148 The University of Kansas, Lawrence, USA; 149 Kansas State University, Manhattan, USA; 150 Lawrence Livermore National Laboratory, Livermore, USA; 151 University of Maryland, College Park, USA; 152 Massachusetts Institute of Technology, Cambridge, USA; 153 University of Minnesota, Minneapolis, USA; 154 University of Mississippi, Oxford, USA; 155 University of Nebraska-Lincoln, Lincoln, USA; 156 State University of New York at Buffalo, Buffalo, USA; 157 Northeastern University, Boston, USA; 158 Northwestern University, Evanston, USA; 159 University of Notre Dame, Notre Dame, USA; 160 The Ohio State University, Columbus, USA; 161 Princeton University, Princeton, USA; 162 University of Puerto Rico, Mayagüez, USA; 163 Purdue University, West Lafayette, USA; 164 Purdue University Calumet, Hammond, USA; 165 Rice University, Houston, USA; 166 University of Rochester, Rochester, USA; 167 Rutgers, The State University of New Jersey, Piscataway, USA; 168 University of Tennessee, Knoxville, USA; 169 Texas A&M University, College Station, USA; 170 Texas Tech University, Lubbock, USA; 171 Vanderbilt University, Nashville, USA; 172 University of Virginia, Charlottesville, USA; 173 Wayne State University, Detroit, USA; 174 University of Wisconsin-Madison, Madison, WI USA; 175 CERN, Geneva, Switzerland

## Abstract

A measurement of the double-differential inclusive jet cross section as a function of jet transverse momentum $p_{\mathrm {T}}$ and absolute jet rapidity $|y |$ is presented. The analysis is based on proton–proton collisions collected by the CMS experiment at the LHC at a centre-of-mass energy of 13$\,\text {TeV}$. The data samples correspond to integrated luminosities of 71 and 44$\,\text {pb}^\text {-1}$ for $|y |<3$ and $3.2<|y |<4.7$, respectively. Jets are reconstructed with the anti-$k_{\mathrm {t}}$ clustering algorithm for two jet sizes, *R*, of 0.7 and 0.4, in a phase space region covering jet $p_{\mathrm {T}}$ up to 2$\,\text {TeV}$ and jet rapidity up to $|y |$ = 4.7. Predictions of perturbative quantum chromodynamics at next-to-leading order precision, complemented with electroweak and nonperturbative corrections, are used to compute the absolute scale and the shape of the inclusive jet cross section. The cross section difference in *R*, when going to a smaller jet size of 0.4, is best described by Monte Carlo event generators with next-to-leading order predictions matched to parton showering, hadronisation, and multiparton interactions. In the phase space accessible with the new data, this measurement provides a first indication that jet physics is as well understood at $\sqrt{s}=13\,\text {TeV}$ as at smaller centre-of-mass energies.

## Introduction

Quantum chromodynamics (QCD) is the fundamental theory describing strong interactions among partons, $\mathrm i.e.$quarks and gluons. Inclusive jet production ($\mathrm {p}\,+\,\mathrm {p}\rightarrow \text {jet}\,+\, \mathrm {X}$) is a key process to test predictions of perturbative QCD (pQCD) over a wide region in phase space. To compare with measurements, the parton-level calculations must be complemented with corrections for nonperturbative (NP) effects that involve the modeling of hadronisation (HAD) and multiparton interactions (MPI). Previous measurements at the CERN LHC have been carried out by the ATLAS and CMS Collaborations at centre-of-mass energies $\sqrt{s} = 2.76\,\text {TeV}$ [1, 2], 7$\,\text {TeV}$  [3–7], and at lower $\sqrt{s}$ by experiments at other hadron colliders [8–12]. The measurements at 2.76 and 7$\,\text {TeV}$ centre-of-mass energies were found to be in agreement with calculations at next-to-leading order (NLO) in the strong coupling constant $\alpha _S$ over a wide range of jet transverse momentum $p_{\mathrm {T}}$ and rapidity *y*. With the latest data from the LHC Run 2, these tests of pQCD are extended to cover the new energy regime of $\sqrt{s}=13\,\text {TeV}$.

In this paper, a measurement of the double-differential inclusive jet cross section is presented as a function of the jet $p_{\mathrm {T}}$ and absolute jet rapidity $|y |$. The jets are clustered with the anti-$k_{\mathrm {t}}$ jet algorithm [13] as implemented in the FastJet library [14]. Two jet sizes *R* are used: the larger value $R=0.7$ corresponds to the standard jet size chosen in most QCD jet analyses made by the CMS Collaboration because it favourably compares to fixed-order predictions [15]. A second, smaller value of *R* emphasizes different aspects of perturbative and nonperturbative QCD and permits complementary tests to be performed [16–18]. Moreover, the choice of $R=0.4$ as a new CMS default jet size that replaces the previous one of 0.5 in LHC Run 1 analyses will allow direct comparisons between jet measurements made by ATLAS and CMS.

The proton–proton collision data were recorded by the CMS experiment at a centre-of-mass energy of 13$\,\text {TeV}$ in 2015. The data samples correspond to integrated luminosities of 71 and 44$\,\text {pb}^{-1}$ for ranges in rapidity of $|y | < 3$ and $3.2< |y | < 4.7$, respectively. The smaller amount of data for the forward rapidity range is explained by more difficult operating conditions at the very start of data taking, which reduced the event sample certified for physics analyses. The results are compared to fixed-order predictions at NLO precision, complemented with electroweak and nonperturbative corrections, and to predictions of various Monte Carlo (MC) event generators that combine leading-order (LO) or NLO pQCD with the modeling of parton showers (PS), HAD, and MPI.

## The CMS detector

The central feature of the CMS apparatus is a superconducting solenoid of 6 m internal diameter, providing a magnetic field of 3.8 T. Within the solenoid volume are a silicon pixel and strip tracker, a lead tungstate crystal electromagnetic calorimeter (ECAL), and a brass and scintillator hadron calorimeter (HCAL), each composed of a barrel and two endcap sections. Forward calorimeters extend the pseudorapidity ($\eta$) coverage provided by the barrel and endcap detectors to the region $3.0< |y | < 5.2$. Muons are measured in gas-ionisation detectors embedded in the steel flux-return yoke outside the solenoid. In the region $|\eta | < 1.74$, the HCAL cells have widths of 0.087 in $\eta$ and 0.087 radians in azimuth ($\phi$). In the $\eta$-$\phi$ plane, and for $|\eta | < 1.48$, the HCAL cells map onto $5 \times 5$ ECAL crystals arrays to form calorimeter towers projecting radially outwards from close to the nominal interaction point. At larger values of $|\eta |$, the size in rapidity of the towers increases and the matching ECAL arrays contain fewer crystals. Within each tower, the energy deposits in ECAL and HCAL cells are summed to define the calorimeter tower energies, subsequently used to provide the energies and directions of hadronic jets. The particle-flow (PF) event algorithm [19, 20] reconstructs and identifies each individual particle with an optimised combination of information from the various elements of the CMS detector. The energy of photons is directly obtained from the ECAL measurement. The energy of electrons is determined from a combination of the electron momentum at the primary interaction vertex as determined by the tracker, the energy of the corresponding ECAL cluster, and the energy sum of all bremsstrahlung photons spatially compatible with originating from the electron track. The momentum of muons is obtained from the curvature of the corresponding track. The energy of charged hadrons is determined from a combination of their momenta measured in the tracker and the matching ECAL and HCAL energy deposits, corrected for zero-suppression effects and for the response function of the calorimeters to hadronic showers. Finally, the energy of neutral hadrons is obtained from the corresponding ECAL and HCAL energy. When combining information from the entire detector, the jet energy resolution typically amounts to 15 % at 10$\,\text {GeV}$, 8 % at 100$\,\text {GeV}$, and 4 % at 1$\,\text {TeV}$, to be compared to about 40, 12, and 5 % obtained when the ECAL and HCAL alone are used. A more detailed description of the CMS detector, together with a definition of the coordinate system used and the relevant kinematic variables, can be found in Ref. [21].

## Event selection and jet reconstruction

The measurement is based on data samples collected with single-jet high-level triggers (HLT) [22]. Eight single-jet HLT paths are considered, seeded by Level 1 triggers based on calorimetric information. They require, in the full rapidity coverage of the CMS detector, at least one jet in each event with $p_{\mathrm {T}} > 60$, 80, 140, 200, 260, 300, 400, or 450$\,\text {GeV}$. All triggers, except the one with the highest threshold, are prescaled. The relative efficiency of each trigger is estimated using lower-$p_{\mathrm {T}}$-threshold triggers, and found to exceed 99 % in the $p_{\mathrm {T}}$ regions shown in Table 1. The absolute trigger efficiency is measured using a tag and probe method [23] based on events selected with a single-jet trigger threshold of 40$\,\text {GeV}$, a back-to-back dijet system, and a probe jet matched to a HLT trigger object. This trigger has an efficiency greater than 99 % for selecting an event with a jet of $p_{\mathrm {T}} > 80\,\text {GeV}$.

**Table 1:** Trigger regions defined as ranges of the leading jet $p_{\mathrm {T}}$ in each event for all single-jet triggers used in the inclusive jet cross section measurement

HLT path	$p_{\mathrm {T}}$ range ($\text {GeV}$)
PFJet$\_$60	114–133
PFJet$\_$80	133–220
PFJet$\_$140	220–300
PFJet$\_$200	300–430
PFJet$\_$260	430–507
PFJet$\_$300	507–638
PFJet$\_$400	638–737
PFJet$\_$450	${>}737$

The main physics objects in this analysis are PF jets, reconstructed by clustering the Lorentz vectors of the PF candidates with the anti-$k_{\mathrm {t}}$ (AK) clustering algorithm for the two jet sizes $R = 0.7$ and 0.4 that will be referred to as AK7 and AK4, respectively. In order to reduce the contribution to the reconstructed jets from additional proton–proton interactions within the same or neighbouring bunch crossings (pileup), the technique of charged hadron subtraction [24] is used. Pileup produces unwanted calorimetric energy depositions and additional tracks. The charged hadron subtraction reduces these effects by removing charged particles that originate from pileup vertices. The average number of pileup interactions observed in these data is ${\approx }19$. During data collection the LHC operated with a 50 ns bunch spacing.

Reconstructed jets require small energy corrections to account for residual nonuniformities and nonlinearities in the detector response. Jet energy scale (JES) [23] corrections are obtained using simulated events, generated with pythia8.204 [25] with tune CUETM1 [26] and processed through the CMS detector simulation, and in situ measurements with dijet, photon+jet, and $\mathrm{{Z}}$+jet events. An offset correction is applied to account for the extra energy clustered into jets due to the contribution of neutral particles produced by additional pileup interactions within the same or neighbouring bunch crossings.

The JES correction, applied as a multiplicative factor to the jet four-momentum vector, depends on the jet $\eta$ and $p_{\mathrm {T}}$ values. The typical correction is about 10 % for a central jet with a $p_{\mathrm {T}}$ of 100$\,\text {GeV}$, and decreases with increasing $p_{\mathrm {T}}$.

Events are required to have at least one primary vertex (PV). If more than one primary vertex is present, the vertex with the highest sum of the squared $p_{\mathrm {T}}$ of the associated tracks is selected. This selected vertex is required to be reconstructed from at least five charged-particle tracks and must satisfy a set of quality requirements, including $|z_\mathrm {PV} | < 24\,\mathrm{cm}$ and $\rho _{\mathrm {PV}} < 2\,\mathrm{cm}$, where $z_{\mathrm {PV}}$ and $\rho _{\mathrm {PV}}$ are the longitudinal and transverse distances of the primary vertex from the nominal interaction point in the CMS detector. Jets with $p_{\mathrm {T}} > 114\,\text {GeV}$ are grouped in seven different $|y |$ bins. Additional selection criteria are applied to each event to remove spurious jet-like signatures originating from isolated noise patterns in certain HCAL regions. To suppress noise patterns, tight identification criteria are applied [27]: each jet should contain at least two particles, one of which is a charged hadron, and the jet energy fraction carried by neutral hadrons and photons should be less than 90 %. These criteria have an efficiency greater than 99 % for genuine jets.

## Measurement of the double-differential inclusive jet cross section

The double-differential inclusive jet cross section is defined as

1 $$\begin{aligned} \frac{\mathrm{d}^2\sigma }{\mathrm{d}p_{\mathrm {T}} \mathrm{d}y} = \frac{1}{\epsilon \mathcal {L}}\,\frac{N_\mathrm {j}}{\Delta p_{\mathrm {T}} \Delta y}, \end{aligned}$$

where $\mathcal {L}$ is the integrated luminosity, $N_\mathrm {j}$ is the number of jets in a bin of a width $\Delta p_{\mathrm {T}}$ in transverse momentum and $\Delta y$ in rapidity, and $\epsilon$ is the product of the trigger and jet selection efficiencies, which is greater than 99 %. The phase space in rapidity is subdivided into six bins from $y=0$ to $|y |=3$ with $|\Delta y | = 0.5$, and one bin from $|y |=3.2$ to 4.7, the forward rapidity region. The bin width in $p_{\mathrm {T}}$ is chosen in such a way that bin-to-bin migrations due to detector resolution are less than 50 %. In each bin, the statistical uncertainty is derived through the formula $\sqrt{{(4-3f)/(2-f)}}\sqrt{{N_{\text {jets}}}}$, where *f* corresponds to the fraction of events which contribute with exactly one jet in the bin [6]. This procedure corrects for possible multiple entries per event. The fraction *f* is typically larger than 95 % in the entire phase-space considered, thus the correction is small.

The double-differential inclusive jet cross section is corrected for the detector resolution and unfolded to the stable particle level [28]. In this way, a direct comparison of this measurement to results from other experiments and to QCD predictions is possible. Particles are considered stable if their mean path length $c\tau$ is greater than 10 mm.

The unfolding procedure is based on the iterative d’Agostini method [29], as implemented in the RooUnfold software package [30], using a response matrix that maps the predicted distribution onto the measured one. The response matrix is derived from a simulation, that uses the theoretically predicted spectrum as input and introduces smearing effects by taking into account the jet $p_{\mathrm {T}}$ resolution. The predicted spectrum is evaluated from fixed-order calculations based on the NLOJet++ v4.1.13 program [31, 32] within the framework of the fastNLO v2.3.1 package [33], using the CT14 [34] parton distribution functions (PDF). More details are presented in Sect. 5.1. The jet $p_{\mathrm {T}}$ resolution is evaluated with the CMS detector simulation based on Geant4  [35] using a QCD simulation from pythia8 with tune CUETM1, after correcting for the residual differences between data and simulation [23]. The unfolded distributions differ from the distributions at detector level by 5–20 %. The unfolding procedure can turn statistical fluctuations of the measured spectra into correlated patterns among the neighbouring bins. It has been verified that such effects are always within the statistical uncertainties of the unfolded distributions, which are larger than those of the detector-level distributions. The iterative unfolding procedure is regularized by limiting the number of iterations to four in each rapidity bin.

The main systematic uncertainties for the jet cross section measurements arise from the JES calibration and from the uncertainty in the integrated luminosity. The JES uncertainty, evaluated separately for AK7 and AK4 jets, is 1–3 % in the central region ($|y | < 2$) and increases to 7–8 % in the forward rapidity region ($3.2< |y | < 4.7$) [23]. The JES uncertainty also includes the uncertainty carried by the charged hadron subtraction. The resulting uncertainties in the double-differential inclusive jet cross section range between 8 % at central rapidities and low $p_{\mathrm {T}}$ to 65 % at forward rapidities and the highest $p_{\mathrm {T}}$. The uncertainty in the integrated luminosity (2.7 % [36]) propagates directly to the cross section.

The unfolding procedure is affected by uncertainties in the jet energy resolution (JER) parametrisation. Alternative response matrices are used to unfold the measured spectra. They are built by varying the JER parameters within their uncertainties [23]. The JER uncertainty introduces a 1–2 % uncertainty in the measured cross section. The model dependence of the theoretical $p_{\mathrm {T}}$ spectrum also affects the response matrix and thus the unfolding, but this uncertainty has negligible effects on the cross section measurement. The model dependence is checked using various PDF sets to calculate the theoretical $p_{\mathrm {T}}$ spectrum.

Finally, an uncertainty of 1 % is assigned to the cross section to account for residual effects of small inefficiencies from jet identification [15]. The total experimental systematic uncertainty of the measured cross section is obtained by summing in quadrature the individual contributions from JES, luminosity, JER, and jet identification uncertainties.

## Theoretical predictions

### Predictions from fixed-order calculations in pQCD

**Fig. 1:**
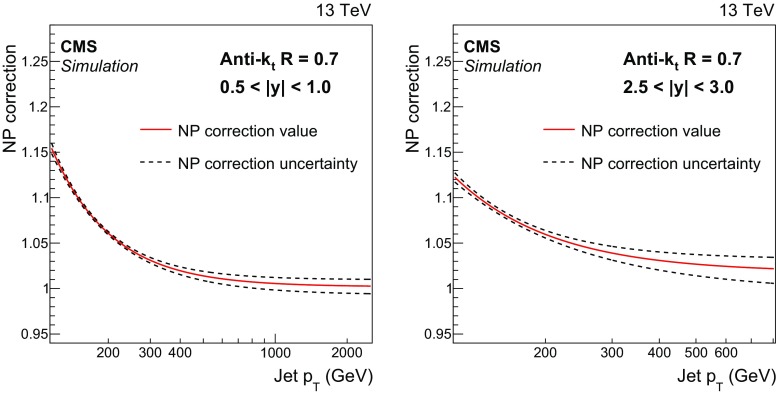
Fits to the nonperturbative corrections obtained for inclusive AK7 jet cross sections as a function of jet $p_{\mathrm {T}}$ for two rapidity bins: $0.5< |y | < 1.0$ (*left*) and $2.5< |y | < 3.0$ (*right*). The *dotted lines* represent the uncertainty bands, which are evaluated by fitting the envelopes of the predictions of the different generators used

**Fig. 2:**
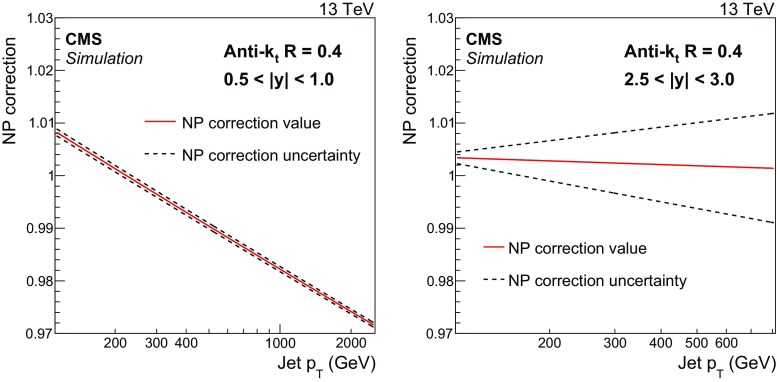
Fits to the nonperturbative corrections obtained for inclusive AK4 jet cross sections as a function of jet $p_{\mathrm {T}}$ for two rapidity bins: $0.5< |y | < 1.0$ (*left*) and $2.5< |y | < 3.0$ (*right*). The *dotted lines* represent the uncertainty bands, which are evaluated by fitting the envelopes of the predictions of the different generators used

**Fig. 3:**
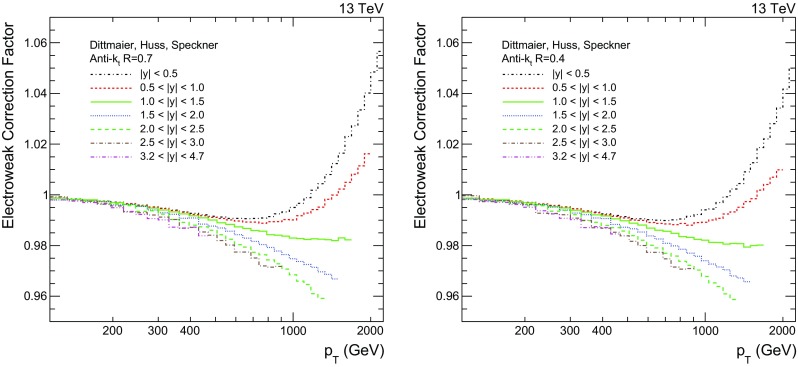
Electroweak correction factors for the seven rapidity bins for the AK7 (*left*) and AK4 (*right*) jets as a function of jet $p_{\mathrm {T}}$

The theoretical predictions for the jet cross section are calculated at NLO accuracy in pQCD and are evaluated by using NLOJet++ within the framework of fastNLO. The cross sections are calculated at NLO for single inclusive jet production. The renormalisation and the factorisation scales ($\mu _r$ and $\mu _f$) are chosen to be equal to the jet $p_{\mathrm {T}}$. Five quarks are assumed to be massless in the calculation, which is performed using four different PDF sets with NLO accuracy: CT14 [34], HERAPDF1.5 [37], MMHT2014 [38], and NNPDF3.0 [39], with the default values of the strong coupling $\alpha _S(M_{\mathrm{{Z}}}) =0.1180$, 0.1176, 0.1200, and 0.1180, respectively.

The theoretical uncertainties are evaluated as the quadratic sum of the scale, PDF, $\alpha _S$, and NP uncertainties. The scale uncertainty is calculated by varying $\mu _r$ and $\mu _{f}$ in the following six combinations: ($\mu _r$/$p_{\mathrm {T}}$, $\mu _f$/$p_{\mathrm {T}}$) = (1/2,1/2), (1/2,1), (1,1/2), (1,2), (2,1) and (2,2). The (asymmetric) scale uncertainty is determined through the maximal upwards and downwards deviations with respect to cross sections obtained with the default setting. The PDF and $\alpha _S$ uncertainties are calculated according to the prescription of CT14 at the 90 % confidence level and scaled down to a 68.3 % confidence level.

The impact of NP effects, i.e. MPI and HAD effects, is evaluated by using samples obtained from different MC event generators with a simulation of PS and MPI contributions. The following MC event generators are used to estimate the NP corrections: LO pythia8 with tune CUETM1, LO herwig++ 2.7.0 [40] with tunes UE-EE-5C [41] and CUETS1 [26], and NLO powheg  [42–44]. The matrix element calculation performed with powheg is interfaced to pythia8 with three different tunes (CUETS1-CTEQ6L1, CUETS1-HERAPDF, and CUETM1) for the simulation of the underlying-event (UE) contributions. The cross section ratios between a nominal event generation interfaced to the simulation of UE contributions, and a sample without HAD and MPI effects are taken as correction separately in each considered rapidity range. In a compact formulation, the NP correction factors can be defined as

2 $$\begin{aligned} C^{\mathrm {\tiny {NP}}}= \frac{ \mathrm{d}\sigma ^{\mathrm {\tiny {PS+HAD+MPI}}} / \mathrm{d}p_{\mathrm {T}}}{ \mathrm{d}\sigma ^{\mathrm {\tiny {PS}}} / \mathrm{d}p_{\mathrm {T}}}, \end{aligned}$$

where $\sigma ^{\mathrm {\tiny {PS+HAD+MPI}}}$ is the cross section obtained with an MC sample simulating the contribution of PS, HAD, and MPI, while $\sigma ^{\mathrm {\tiny {PS}}}$ includes only PS effects. Corrections obtained with various NLO and LO event generators are evaluated separately for the AK7 and AK4 jets. The average of the results from the NLO and LO event generators defines the central value of the NP corrections, which are fitted to a power-law function in jet $p_{\mathrm {T}}$. The uncertainty in the NP corrections are evaluated by fitting the upper and lower values of the predictions of the different generators. The combinations of PDF sets, matrix element calculations, and UE tunes used to evaluate the NP corrections are validated on UE, minimum bias and jet variables, and they are able to reproduce a wide set of observables [26]. The NP corrections are shown in Figs. 1 and 2, respectively, for AK7 and AK4 jets in a central ($0.5< |y | < 1.0$) and a forward rapidity bin ($2.5< |y | < 3.0$).

The NP corrections for the AK7 jets are $\approx$15 % (13 %) for $p_{\mathrm {T}}$ $\sim$ 114$\,\text {GeV}$ in the region $0.5<|y |<1.0$ ($2.5<|y |<3.0$) and decrease rapidly for increasing $p_{\mathrm {T}}$, flattening at values of ${\approx }1$ for $p_{\mathrm {T}} \sim 200$–300$\,\text {GeV}$, depending on the considered rapidity range. Because of the smaller cone size, AK4 jets are less affected by the MPI and HAD effects. In particular, the additional energy produced by MPI shrinks for decreasing radii R, while the out-of-cone losses due to HAD effects increase for smaller radii R. These two effects are responsible for NP corrections that fall below 1 for AK4 jets with $p_{\mathrm {T}} >200\,\text {GeV}$ at central rapidity. The NP corrections for AK4 jets are very close to unity in the phase space considered. For both cone sizes, the uncertainty assigned to the NP corrections is of the order of 1–2 %.

**Fig. 4:**
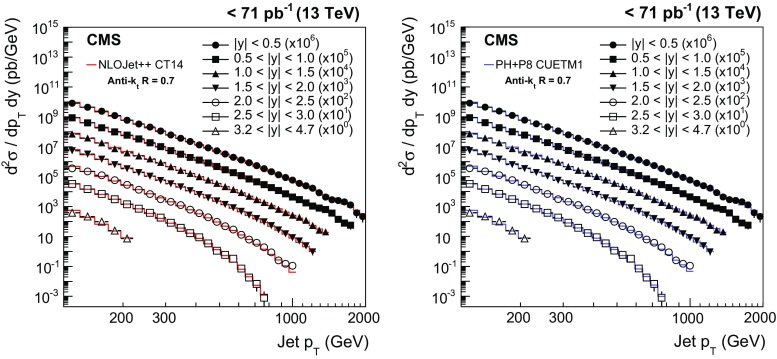
Double-differential inclusive jet cross section as function of jet $p_{\mathrm {T}}$. On the *left*, data (*points*) and predictions from NLOJet++ based on the CT14 PDF set corrected for the NP and electroweak effects (*line*) are shown. On the *right*, data (*points*) and predictions from powheg (PH) + pythia8 (P8) with tune CUETM1 (*line*) are shown. Jets are clustered with the anti-$k_{\mathrm {t}}$ algorithm ($R = 0.7$)

**Fig. 5:**
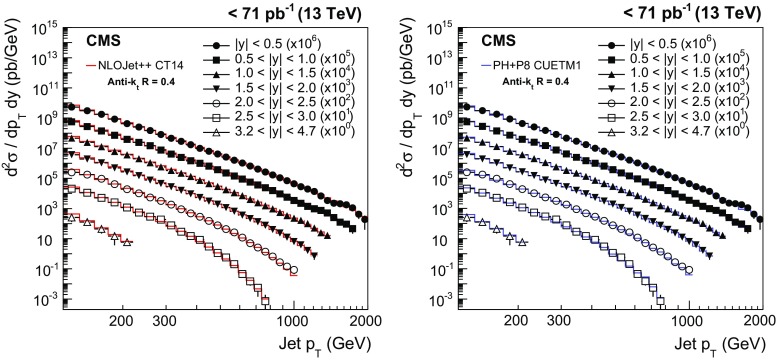
Double-differential inclusive jet cross section as function of jet $p_{\mathrm {T}}$. On the* left*, data (*points*) and predictions from NLOJet++ based on the CT14 PDF set corrected for the NP and electroweak effects (*line*) are shown. On the* right*, data (*points*) and predictions from powheg (PH) + pythia8 (P8) with tune CUETM1 (*line*) are shown. Jets are clustered with the anti-$k_{\mathrm {t}}$ algorithm ($R = 0.4$)

Electroweak effects, which arise from the virtual exchanges of massive gauge W and Z bosons, become sizable at high jet $p_{\mathrm {T}}$ and central rapidity. Corrections to electroweak effects are shown in Fig. 3 for both AK7 and AK4 jets [45]. They range between 0.96 and 1.05, depending on the jet $p_{\mathrm {T}}$ and rapidity, and are less than 3 % for $p_{\mathrm {T}} <1\,\text {TeV}$ and very similar between the two cone sizes. For jet measurements performed at a centre-of-mass energy of 7$\,\text {TeV}$  [46], electroweak corrections of 10–15 % are observed for jet $p_{\mathrm {T}} >1\,\text {TeV}$ in the $|y |<1.0$ range, decreasing below 2 % for lower $p_{\mathrm {T}}$, independent of the jet rapidity. Electroweak corrections are applied to the NLOJet++ predictions in a similar manner to the NP contributions.

### Predictions from fixed-order calculations matched to parton shower simulations

The predictions from different MC event generators are compared to data. The herwig++ and the pythia8 event generators are considered. Both of them are based on an LO $2\rightarrow 2$ matrix element calculation. The pythia8 event generator simulates parton showers ordered in $p_{\mathrm {T}}$ and uses the Lund string model [47] for HAD, while herwig++ generates parton showers through angular-ordered emissions and uses a cluster fragmentation model [48] for HAD. The contribution of MPI is simulated in both pythia8 and herwig++ . In particular, pythia8 applies a model [49] where MPI are interleaved with parton showering, while herwig++ models the overlap between the colliding protons through a Fourier transform of the electromagnetic form factor, which plays the role of an effective inverse proton radius. Depending on the amount of proton overlap, the contribution of generated MPI varies in the simulation. The MPI parameters of both generators are tuned to measurements in proton–proton collisions at the LHC [26], while the HAD parameters are determined from fits to LEP data. For pythia8, the CUETM1 tune, which is based on NNPDF2.3LO [50, 51], is considered, while herwig++ uses the CUETS1 tune [26], based on the CTEQ6L1 PDF set [52].

Predictions based on NLO pQCD are also considered using the powheg package matched to pythia8 parton showers and including a simulation of MPI. The powheg sample uses the CT10nlo PDF set [53]. Various tunes in pythia8 are used for the UE simulation, which differ in the choice of the PDF set and the HAD parameters: the CUETM1, and tunes CUETS1-CTEQL1 and CUETS1-HERAPDF, which use the CTEQ6L1 and the HERAPDF1.5LO [54] PDF sets, respectively. The HAD parameters for the CUETM1 tune are taken from the Monash tune [55], while the 4C tune provides these in both CUETS1 tunes. All these combinations of powheg matrix element and UE-simulation tunes reproduce with very high precision the UE and jet observables at various collision energies [26].

## Comparison of theoretical predictions and data

Figures 4 and 5 show the double-differential inclusive jet cross section measurements, presented as a function of $p_{\mathrm {T}}$ for seven $|y |$ ranges, after unfolding for detector effects, using the anti-$k_{\mathrm {t}}$ algorithm with $R = 0.7$ and 0.4, respectively. The measurements are compared to the NLOJet++ predictions based on the CT14 PDF set, corrected for NP and electroweak effects (left), and to the predictions from powheg + pythia8 with tune CUETM1 (right). The data are consistent with the predictions over a wide range of jet $p_{\mathrm {T}}$ from 114$\,\text {GeV}$ up to 2$\,\text {TeV}$.

**Fig. 6:**
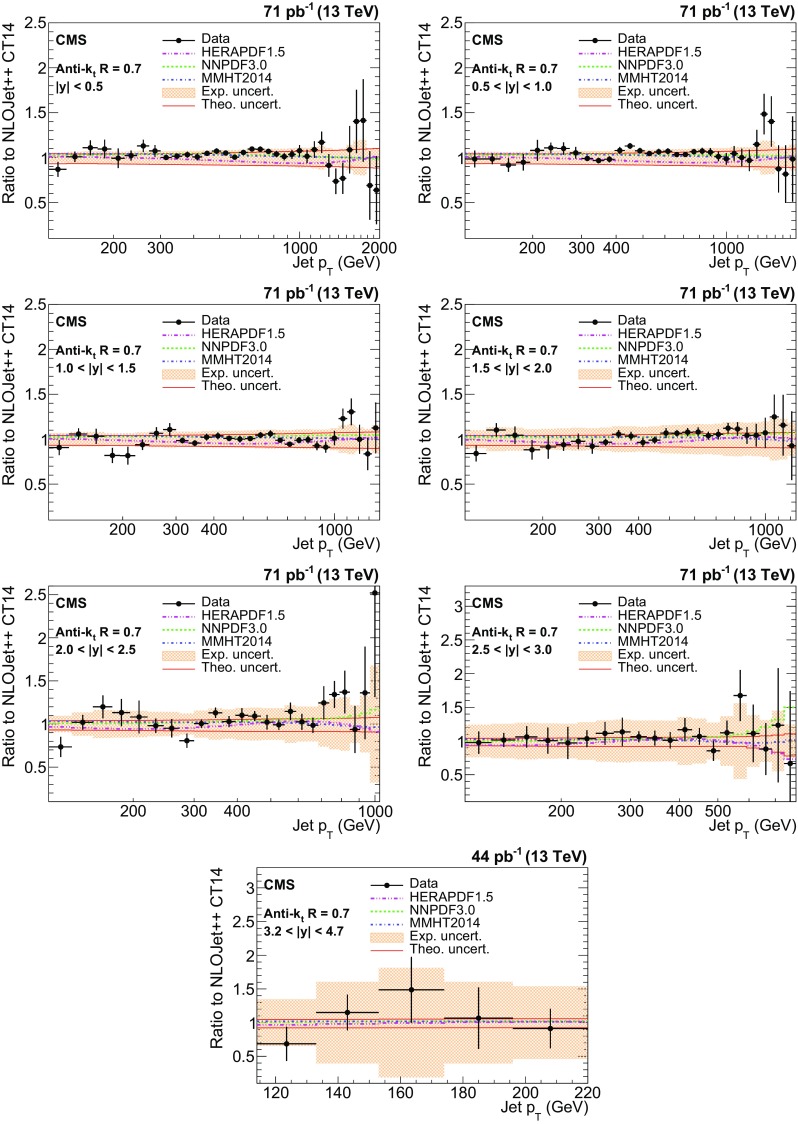
Ratio of measured values to theoretical prediction from NLOJet++ using the CT14 PDF set and corrected for the NP and electroweak effects. Predictions employing three other PDF sets are also shown for comparison. Jets are clustered with the anti-$k_{\mathrm {t}}$ algorithm with a distance parameter of 0.7. The *error bars* correspond to the statistical uncertainties of the data and the *shaded bands* to the total experimental systematic uncertainties

**Fig. 7:**
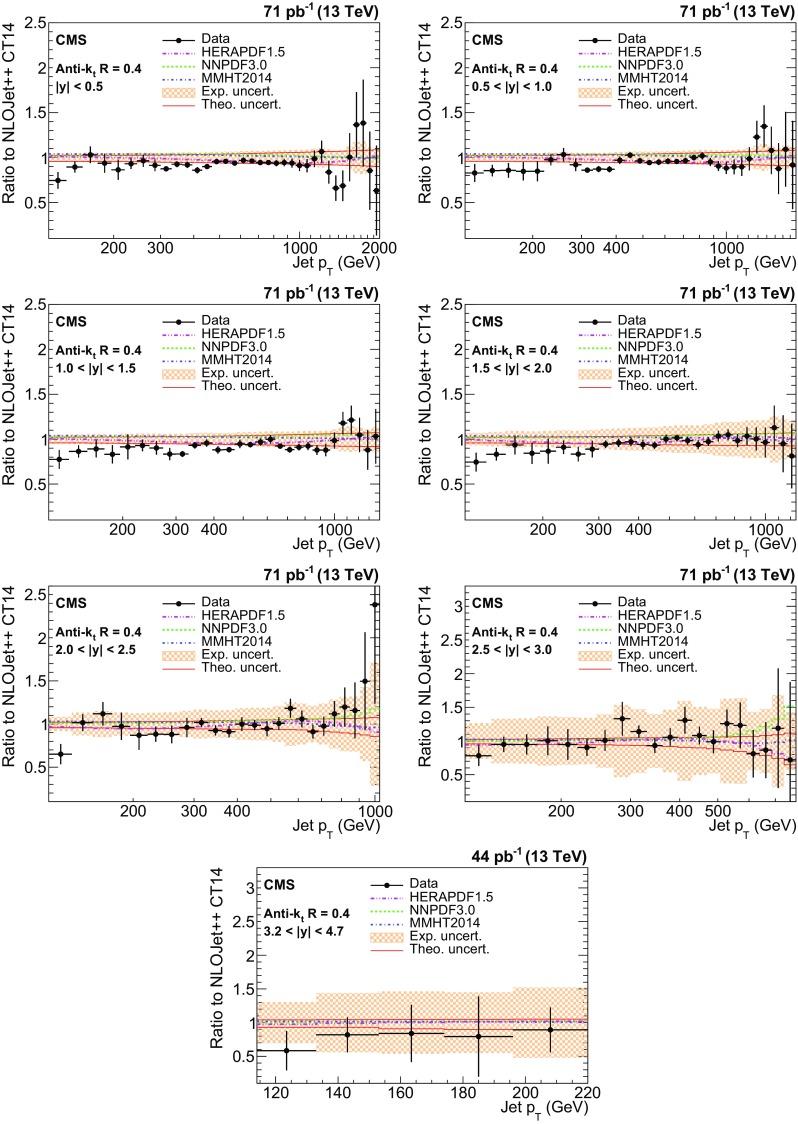
Ratio of measured values to theoretical prediction from NLOJet++ using the CT14 PDF set and corrected for the NP and electroweak effects. Predictions employing three other PDF sets are also shown for comparison. Jets are clustered with the anti-$k_{\mathrm {t}}$ algorithm with a distance parameter of 0.4. The *error bars* correspond to the statistical uncertainties of the data and the *shaded bands* to the total experimental systematic uncertainties

The ratios of data over the NLOJet++ predictions using the CT14 PDF set are shown in Fig. 6 for the AK7 jets. The error bars on the points correspond to the statistical uncertainties, and the shaded bands correspond to the total experimental systematic uncertainties. For comparison, predictions employing three alternative PDF sets are also shown. Figure 7 shows the results for the AK4 jets. Overall, a good agreement within the uncertainties is observed between the data and predictions in the entire kinematic range studied, for both jet cone sizes. However, for $R = 0.4$, the cross sections are systematically overestimated by about 5–10 %, while a better description is provided for jets reconstructed with $R = 0.7$. The relatively poor agreement for $R = 0.4$ is due to PS and soft-gluon resummation contributions, which are missing in fixed-order calculations, and that are more relevant for smaller jet cone sizes because of out-of-cone effects.

**Fig. 8:**
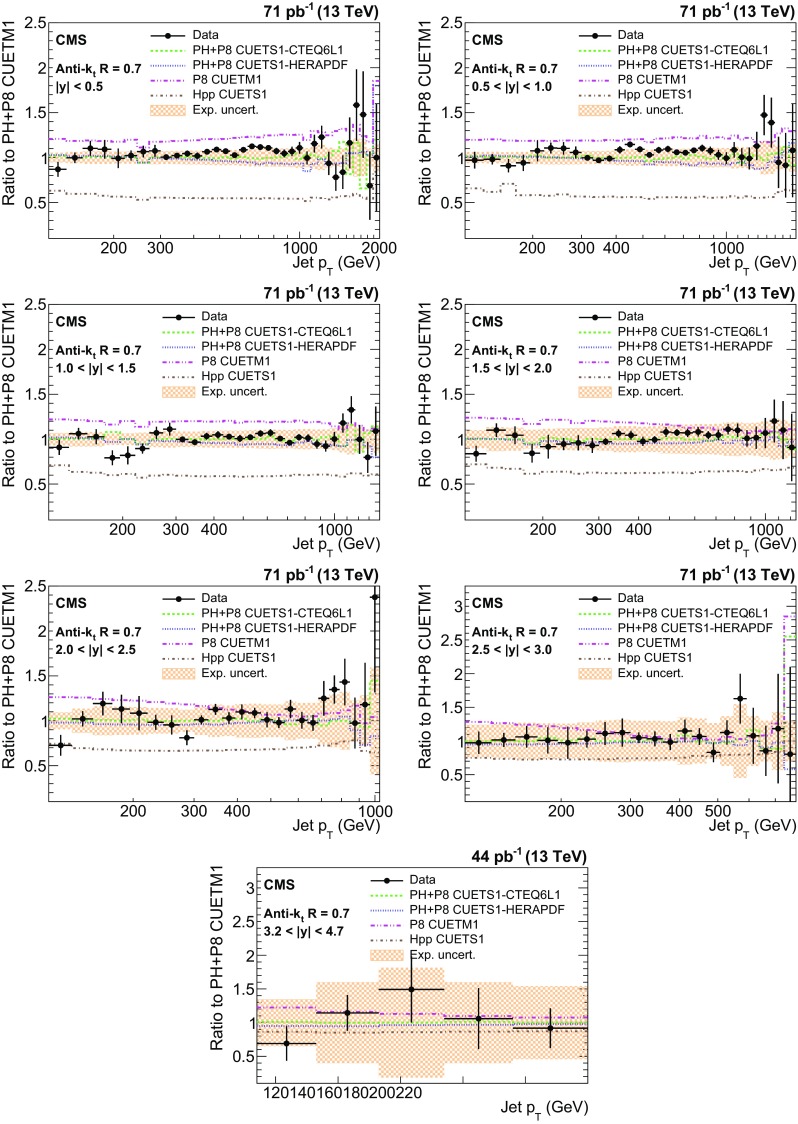
Ratio of measured values to predictions from powheg (PH) + pythia8 (P8) with tune CUETM1. Predictions employing four other MC generators are also shown for comparison, where PH, P8, and Hpp stands for powheg, pythia8, and herwig++ (HPP), respectively. Jets are clustered with the anti-$k_{\mathrm {t}}$ algorithm with a distance parameter of 0.7. The *error bars* correspond to the statistical uncertainties of the data and the *shaded bands* to the total experimental systematic uncertainties

**Fig. 9:**
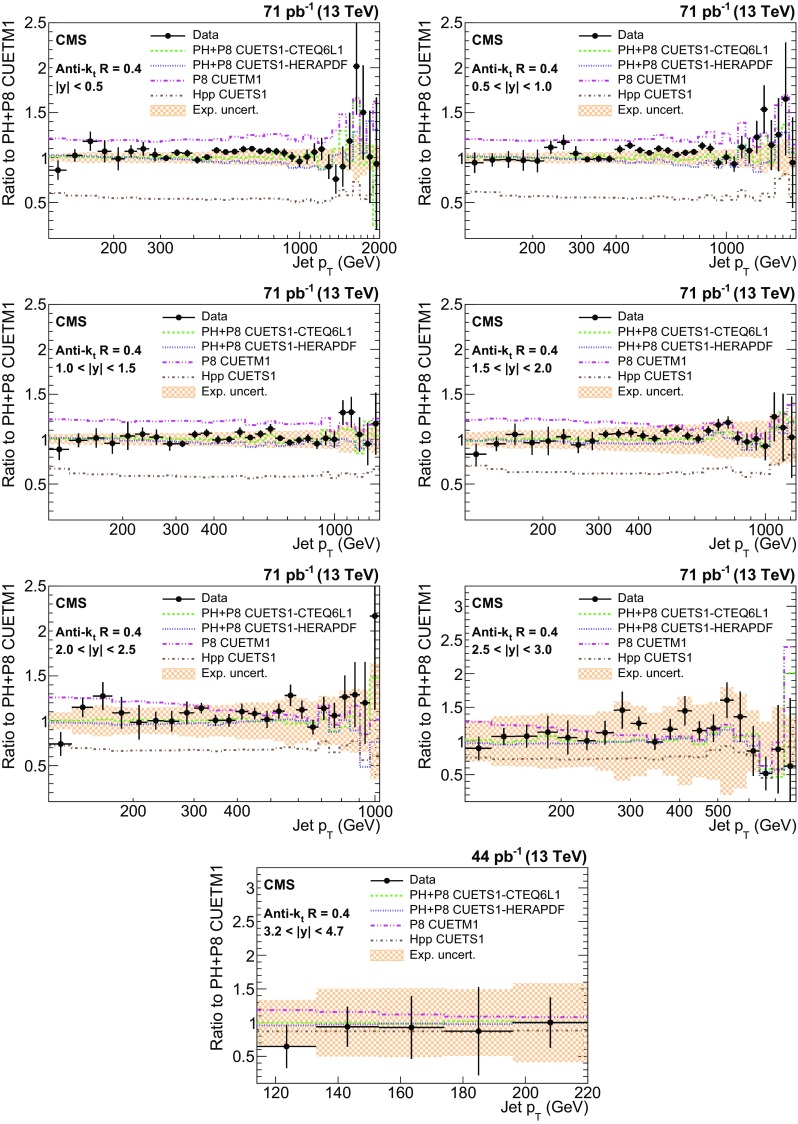
Ratio of measured values to predictions from powheg (PH) + pythia8 (P8) with tune CUETM1. Predictions employing four other MC generators are also shown for comparison, where PH, P8, and Hpp stands for powheg, pythia8, and herwig++ (HPP), respectively. Jets are clustered with the anti-$k_{\mathrm {t}}$ algorithm with a distance parameter of 0.4. The *error bars* correspond to the statistical uncertainties of the data and the *shaded bands* to the total experimental systematic uncertainties

The ratios of data over predictions from powheg + pythia8 with tune CUETM1 are shown in Figs. 8 and 9 for the AK7(AK4) jets. The error bars on the points correspond to the statistical uncertainties and the shaded bands to the total experimental systematic uncertainties. For comparison, four other MC predictions are also shown. There is an overall good level of agreement within the uncertainties between data and predictions from powheg + pythia8 with various tunes for both cone sizes, in the entire kinematic range studied. The agreement of data with pythia8 and herwig++ is poor in absolute scale. The herwig++ event generator shows good agreement with the data in shape for all rapidity bins, while pythia8 agrees well in shape with the data for only $|y | < 1.5$.

## Summary

A measurement of the double-differential cross section as a function of jet $p_{\mathrm {T}}$ and absolute rapidity $|y |$ is presented for two jet sizes $R=0.4$ and 0.7 using data from proton–proton collisions at $\sqrt{s}=13\,\text {TeV}$ collected with the CMS detector. Data samples corresponding to integrated luminosities of 71 and 44$\,\text {pb}^\text {-1}$ are used for absolute rapidities $|y |<3$ and for the forward region $3.2<|y |<4.7$, respectively.

As expected for LO predictions, the MC event generators pythia8 and herwig++ exhibit significant discrepancies in absolute scale with respect to data, which are somewhat more pronounced for the case of herwig++ . In contrast, the shape of the inclusive jet $p_{\mathrm {T}}$ distribution is well described by herwig++ in all rapidity bins. Predictions from pythia8 start deviating from the observed shape as $|y |$ increases.

In the comparison between data and predictions at NLO in perturbative QCD including corrections for nonperturbative and electroweak effects, it is observed that jet cross sections for the larger jet size of $R=0.7$ are accurately described, while for $R=0.4$ theory overestimates the cross section by 5–10 % almost globally. In contrast, NLO predictions matched to parton showers as performed with powheg + pythia8 for two different tunes, perform equally well for both jet sizes. This result is consistent with the previous measurement performed at $\sqrt{s} = 7\,\text {TeV}$ [15], where it was observed that powheg + pythia8 correctly describes the *R* dependence of the inclusive jet cross section, while fixed-order predictions at NLO were insufficient in that respect.

This measurement is a first indication that jet physics is as well understood at $\sqrt{s}=13\,\text {TeV}$ as at smaller centre-of-mass energies in the phase space accessible with the new data.
